# Transitions between the steps of forward and reverse splicing of group IIC introns

**DOI:** 10.1261/rna.075044.120

**Published:** 2020-05

**Authors:** Claire M. Smathers, Aaron R. Robart

**Affiliations:** Department of Biochemistry, West Virginia University, Morgantown, West Virginia 20506, USA

**Keywords:** intron, ribozyme, splicing, retrotransposition

## Abstract

Group II introns are mobile genetic elements that perform both self-splicing and intron mobility reactions. These ribozymes are comprised of a catalytic RNA core that binds to an intron-encoded protein (IEP) to form a ribonucleoprotein (RNP) complex. Splicing proceeds through two competing reactions: hydrolysis or branching. Group IIC intron ribozymes have a minimal RNA architecture, and splice almost exclusively through hydrolysis in ribozyme reactions. Addition of the IEP allows the splicing reaction to form branched lariat RNPs capable of intron mobility. Here we examine ribozyme splicing, IEP-dependent splicing, and mobility reactions of a group IIC intron from the thermophilic bacterium *Thermoanerobacter italicus* (*Ta.it.*I1). We show that *Ta.it.*I1 is highly active for ribozyme activity, forming linear hydrolytic intron products. Addition of purified IEP switches activity to the canonical lariat forming splicing reaction. We demonstrate that the *Ta.it.*I1 group IIC intron coordinates the progression of the forward splicing reaction through a π–π′ interaction between intron domains II and VI. We further show that branched splicing is supported in the absence of the IEP when the π–π′ interaction is mutated. We also investigated the regulation of the two steps of reverse splicing during intron mobility into DNA substrates. Using a fluorescent mobility assay that simultaneously visualizes all steps of intron integration into DNA, we show that completion of reverse splicing is tightly coupled to cDNA synthesis regardless of mutation of the π–π′ interaction.

## INTRODUCTION

Group II introns are self-splicing ribozymes that reside within the genomes of bacteria (Ferat and Michel 1993; Toro 2003) and the organelles of yeast, fungi, algae, and plants (Michel et al. 1989; Fedorova and Zingler 2007; Bonen 2008). Although these RNAs are divergent in primary sequence, they fold into highly conserved structures that catalyze self-excision from precursor transcripts (Qin and Pyle 1998; Toor et al. 2001,^2009^; Michel et al. 2009). In addition to their highly structured RNA machinery, many group II introns also contain an open reading frame encoding an intron-encoded protein (IEP) (Michel and Lang 1985; Mohr et al. 1993; Zimmerly et al. 2001). This multidomain protein assists splicing activity, and remains bound after splicing to form intron lariat-IEP ribonucleoproteins (RNPs). The predominant domain of the IEP is a reverse transcriptase (RT), which allows these RNPs to act as retroelements that invade new DNA locations (for reviews, see Lambowitz and Zimmerly 2004; Lambowitz and Belfort 2015; Zimmerly and Semper 2015).

**FIGURE 1.**
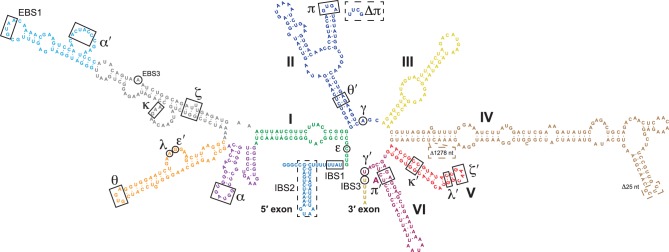
Secondary structure of the *Ta.it.*I1 group IIC intron. Six RNA secondary structure domains are shown radiating from a central hub. Colors of domains match previously published group II intron crystal structures. Domain IV encodes an ORF that was removed for in vitro ribozyme splicing assays; points of deletion are shown. Greek letters indicate RNA tertiary interactions. Mutation of the putative π tetraloop is shown. Exons are recognized by IBS-EBS elements, γ–γ′, and a terminator stem–loop element.

Group II introns are divided into three major classes based on IEP sequence and RNA secondary structural features: IIA, IIB, and IIC (Toor et al. 2001). While all three classes share a general six domain secondary structure radiating from a central wheel (Michel et al. 1989), they have significant structural and functional differences (Fig. 1). Group IIC introns are the smallest and most divergent class of group II introns, missing significant portions of RNA secondary structure compared to IIA and IIB introns (Granlund et al. 2001; Toor et al. 2001). IIC introns also have relaxed sequence requirements for splice site recognition, and utilize a stem–loop structure to decode the 5′ splice site instead of the extensive base-pairing interactions observed in other intron classes (Fig. 1; Toor et al. 2006; Robart et al. 2007; Mohr et al. 2018). Normally, splicing initiates by a bulged adenosine nucleophile in domain VI (DVI) attacking the 5′ splice site, resulting in the formation of branched lariat product (Peebles et al. 1986; Schmelzer and Schweyen 1986; van der Veen et al. 1986). Curiously, almost all IIC introns splice exclusively through hydrolysis in ribozyme reactions to form linear intron products (Toor et al. 2006, 2008, 2010). Biochemical studies have shown that group IIC introns are dependent upon their IEP to switch from hydrolysis to the canonical branched splicing pathway (Robart et al. 2007; Mohr et al. 2018). This suggests that reduction in RNA secondary structure complexity in IIC introns coincides with greater dependence on the IEP for proper positioning of the DVI bulged adenosine in the ribozyme catalytic core.

Structural studies of group IIB introns have revealed that both protein and RNA tertiary interactions play pivotal roles in positioning DVI between the two steps of splicing. In the pre-second step of reverse splicing (analogous to the conformation expected in the first step of forward splicing) DVI is positioned by RNA–RNA interactions with DIC, and protein–RNA interactions with the IEP (Haack et al. 2019). In this conformation, the bulged adenosine is positioned in close proximity to the ribozyme active site. The transition between the first and second step of forward splicing is mediated by two RNA tetraloop interactions between DII and DVI in IIB introns, namely π–π′ and η–η′ (Chanfreau and Jacquier 1996; Robart et al. 2014). This DII–DVI interface has also been observed in cryo-EM studies of a group IIA RNP (Qu et al. 2016). Together these studies support a mechanism by which DVI undergoes a large conformational change. Before the first step of splicing DVI is positioned by both RNA–RNA and IEP–RNA interactions. These interactions hold DVI in close association with DIC in an “up” conformation, positioning the bulged adenosine for attack in the ribozyme active site. During the transition to the second step of splicing, DVI swings to the “down” conformation by engaging the DII–DVI π–π′ and η–η′ tertiary interaction interface. This elegant swinging action removes the first-step splicing branch point from the active site allowing the second step of splicing to proceed. Many group IIA and IIB introns form branched splicing products in the absence of their IEPs; thus, RNA tertiary interactions alone are sufficient to position DVI. In contrast, group IIC introns lack the η–η′ tertiary interaction and strictly require their IEP for these reactions, suggesting that group IIC introns utilize different structural features to coordinate DVI positioning. The overall secondary structure of DII is similar between group IIB and group IIC introns (Toor et al. 2008); however, it is currently unclear if the similar DII secondary structural elements of group IIB and group IIC introns correlate to a common mechanism of splicing.

In this study, we show that a group IIC intron from the thermophilic bacterium *Thermoanerobacter italicus* (*Ta.it.*I1) and its IEP can be readily reconstituted in vitro, and use this system to investigate the transition between the steps of both forward and reverse splicing. We demonstrate that the *Ta.it.*I1 group IIC intron requires a basic amino acid patch within the IEP to promote the formation of branched splicing products, and uses the π–π′ interaction to transition between the steps of the forward splicing reaction. We also find that mutation of the π–π′ interaction allows lariat formation independent of the IEP. We developed a fluorescent assay to investigate *Ta.it.*I1 retromobility into DNA. Using this assay, we show that the engagement of the RT domain of the IEP in cDNA synthesis mediates the transition between the steps of the reverse splicing reaction during *Ta.it.*I1 integration into DNA substrates.

## RESULTS

### The *Ta.it*.I1 ribozyme and IEP are highly active in vitro

**FIGURE 2.**
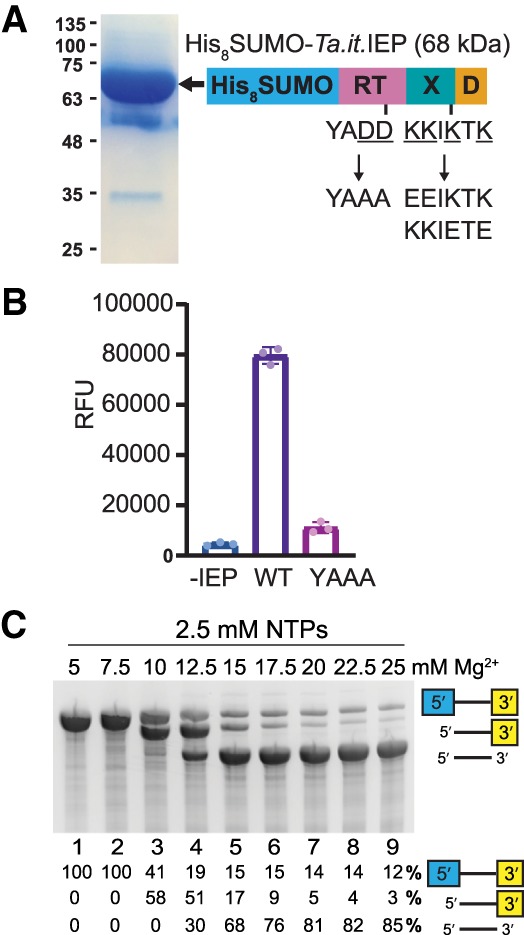
Reconstitution of *Ta.it.*I1 IEP and precatalytic intron RNA. (*A*) Schematic representation of the *Ta.it.*I1 IEP purification construct and SDS-PAGE of purified IEP. Mutations of the IEP used in this study are shown. (*B*) Fluorescent reverse transcription assay of WT and YAAA mutant IEP. (*C*) 4% PAGE analysis of *Ta.it.*I1 intron in vitro transcription products under various [MgCl_2_].

Mobile group II introns use an intron-encoded protein (IEP) to assist ribozyme splicing and facilitate intron mobility into DNA targets. To reconstitute IEP-dependent splicing in vitro, an amino-terminal fusion of His_8_-SUMO-IEP was expressed in *E. coli* by auto-induction (Studier 2005), followed by affinity purification on Ni-agarose resin (Fig. 2A). The eluted protein was highly active for reverse transcriptase activity in assays with poly(rA)·oligo(dT)_18_ substrate using PicoGreen fluorescent incorporation to monitor RT activity (Fig. 2B). As a control, we expressed and purified a mutant version of the IEP where the conserved YADD catalytic motif of the RT domain was mutated to YAAA. Mutation of the tandem aspartic acid residues to alanine perturbs binding of catalytic Mg^2+^ metal ions to inhibit RT activity. The mutant IEP's catalytic activity was significantly reduced, but not completely inactive compared to WT (Fig. 2B). The purified IEP retained activity after removal of the His-SUMO purification tag through proteolytic cleavage by Ulp-1 (SUMO protease).

To reconstitute group II intron in vitro self-splicing reactions, *Ta.it.*I1 intron RNA was prepared by in vitro transcription using T7 RNA polymerase (Wiryaman and Toor 2017). Under standard in vitro transcription conditions, with buffers containing 25 mM MgCl_2_, we observed multiple RNA species migrating at lower molecular weights than the expected precursor product (Fig. 2C, lane 9). These findings demonstrate that the *Ta.it.*I1 intron performs hydrolytic cotranscriptional splicing where a first step water nucleophile produces linear intron. This activity is consistent with previous observations of the ability of group IIC introns to perform hydrolytic splicing in vitro. The identity of the hydrolytic splicing products was confirmed by repeating the transcription with templates varying the length of the 3′ exon (data not shown). Many group II introns have limited activity in vitro, most likely due to RNA misfolding. Thus, the almost complete (88%, Fig. 2C, lane 9) cotranscriptional ribozyme splicing demonstrates that *Ta.it.*I1 RNA efficiently folds into a highly active ribozyme. Development of a custom transcription protocol was necessary for production of precatalytic RNA. We performed transcription under a range of MgCl_2_ concentrations (Fig. 2C). The *Ta.it.*I1 intron spliced to near completion during transcription under conditions containing transcription buffers formulated with >13 mM Mg^2+^ (Fig. 2C, lane 5–9). In vitro transcription conditions of 5 mM MgCl_2_ and 2.5 mM rNTPs were sufficient to prevent cotranscriptional splicing and support activity of T7 RNA polymerase to yield the precatalytic intron population necessary to test self-splicing activity (Fig. 2C, lane 1).

### Reconstitution of IEP-dependent splicing

**FIGURE 3.**
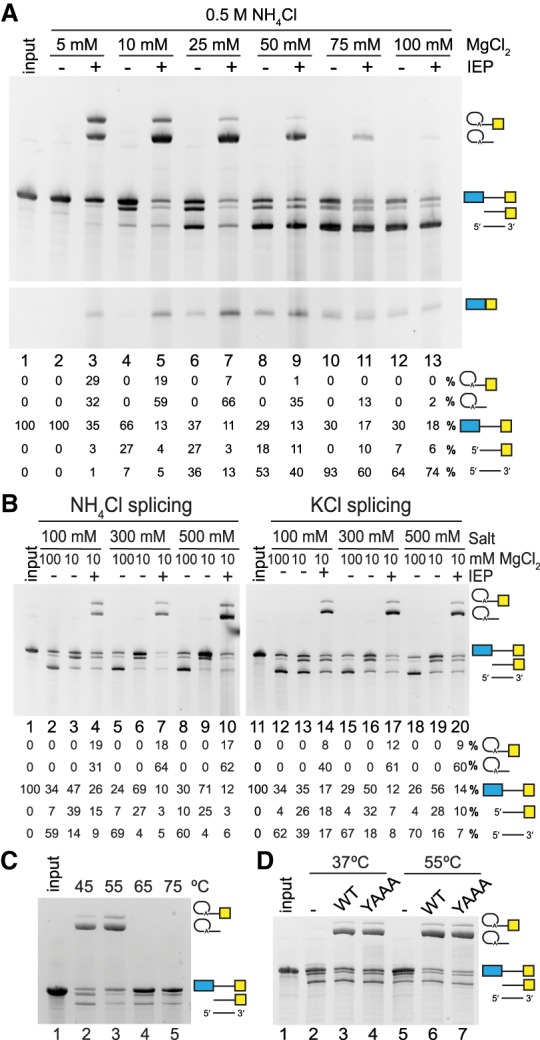
*Ta.it.*I1 in vitro splicing activity. (*A*) *Ta.it.*I1 splicing assay under buffer conditions of 0.5 M NH_4_Cl and [MgCl_2_] ranging from 5–100 mM, with and without addition of IEP at 55°C. (*B*) Comparison of *Ta.it.*I1 splicing in 100–500 mM NH_4_Cl and KCl buffers. (*C*) T*a.it.*I1 intron splicing assay under 0.5 M NH_4_Cl and 10 mM MgCl_2_ buffer at incubation temperatures from 45°C–75°C. (*D*) T*a.it.*I1 intron splicing assay with WT or YAAA mutant IEP.

*Ta.it.*I1 is a group IIC intron, which are highly streamlined introns lacking many hallmark RNA secondary structural features observed in other group II intron families (Fig. 1). To investigate if the reduction in RNA secondary structure correlated to a greater dependence on the IEP to assist lariat formation, we compared splicing in the presence or absence of purified IEP. To examine competition between hydrolytic and branched splicing pathways, we performed a titration of Mg^2+^ ions both in the presence and absence of IEP. In the absence of the IEP, the intron behaves similar to other group IIC introns, exclusively utilizing the hydrolytic splicing pathway resulting in linear intron formation (Fig. 3A, even lanes). Hydrolytic splicing activity was dependent on Mg^2+^ concentration, with maximum activity occurring at [Mg^2+^] above 50 mM. Splicing assays performed in the absence of IEP at 5 mM Mg^2+^ showed no ribozyme activity (Fig. 3A, lane 2); however, upon addition of the IEP, 65% of the intron reacted predominantly through the lariat forming pathway (Fig 3A, lane 3). Thus, formation of an intron-IEP RNP complex switches the splicing reaction from hydrolysis to the canonical lariat forming pathway classically associated with intron splicing reactions. Assays with varying MgCl_2_ concentrations demonstrated that lariat forming efficiency is maximal at 10–25 mM, above which the hydrolytic pathway dominates over intron lariat formation. Splicing was further assayed in both the presence and absence of IEP under a range of 100–500 mM NH_4_Cl at 55°C (Fig. 3B). At 100 mM NH_4_Cl the IEP promoted lariat formation, albeit at lower levels compared to higher salt concentrations (Fig 3B, lanes 4,7,10). Structural analysis of the *Oceanobacillus iheyensis* (*O.i.*) group IIC intron demonstrated that NH_4_Cl and KCl monovalent ions retain the same ribozyme active site architecture (Marcia and Pyle 2012), and it is known that biochemically KCl can promote hydrolytic reactions (Peebles et al. 1987; Jarrell et al. 1988a,b; Daniels et al. 1996). We observed that assays containing KCl promoted a higher level of complete hydrolytic splicing in low MgCl_2_ conditions compared to NH_4_Cl buffers (Fig. 3B, compare lanes 3,6,9 to 13,16,19). The ability of the IEP to stimulate lariat formation over the competing hydrolysis reaction was maintained over temperature ranges from 30°C–55°C when splicing buffer and IEP were preincubated for 5 min before addition of the intron RNA (Fig. 3C). Lariat formation was also supported by the YAAA mutant IEP, demonstrating that a functional RT domain is not required to support group IIC splicing activity (Fig. 3D). Mutation of the RT catalytic domain has been shown previously to support splicing of other group II intron classes (Moran et al. 1995; Cui et al. 2004; Mastroianni et al. 2008)

### IEP and π–π′ interactions are required for branched IIC splicing

**FIGURE 4.**
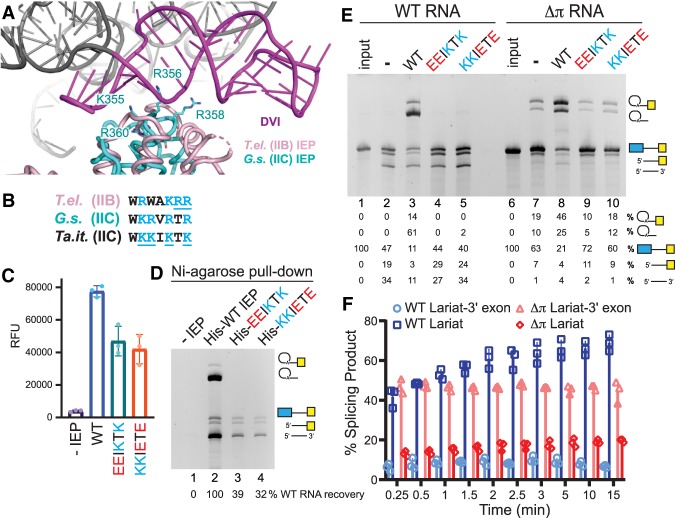
Effects of IEP and π mutations on *Ta.it.*I1 branched splicing. (*A*) Superimposed structures of a cryo-EM IIB RNP structure (PDB 6MEC, magenta) and an active group IIC IEP (PDB 6AR1, teal). (*B*) Alignment of amino acids predicted to interact with DVI. Basic amino acids (blue) underlined were mutated to E in this study (*Ta.it.*) or previously (*T.el.*; Haack et al. 2019). (*C*) RT assay of purified WT and mutant IEPs. (*D*) Pull-down assay of His tagged IEPs against Ni-agarose resin to assess RNP formation. (*E*) WT and mutant IEPs assayed with WT or π mutant *Ta.it.*I1 RNA in 10 mM MgCl_2_ and 0.5 M NH_4_Cl at 55°C. (*F*) Time course of WT and π mutant *Ta.it.*I1 RNA spliced with WT IEP in 10 mM MgCl_2_ and 0.5 M NH_4_Cl at 55°C.

In an effort to examine if a DVI–IEP interaction is required to promote branched IIC intron splicing, we performed least square fit analysis of a IIB RNP cryo-EM structure to an active IIC IEP crystal structure (Emsley et al. 2010; Stamos et al. 2017; Haack et al. 2019). The superimposed structures predict a basic patch of amino acids in group IIC intron IEPs in close proximity to DVI that may function as a DVI binding face (Fig. 4A). Alignments of primary amino acid sequence showed strong conservation between IIB and IIC introns in this predicted DVI–IEP interaction face (Fig. 4B). To test the role of these IEP positions in lariat formation, basic amino acids were mutated to acidic amino acids. Two of these positions were previously shown by similar charge flip mutations to inhibit IIB intron splicing but still support RNP formation (Fig.4B, *T.el.* underlined amino acids; Haack et al. 2019). Mutant IEPs with the sequence changed from KKIKTK to either EEIKTK or KKIETE were expressed and purified. The mutant IEPs retained ∼50% RT activity (Fig. 4C). To assess the ability of the mutant IEPs to form RNPs, we performed a pull-down assay using the His affinity tag in the IEP against Ni-agarose beads blocked with BSA and a non-complementary DNA oligonucleotide. No *Ta.it.*I1 RNA binding was observed in the absence of IEP (Fig. 4D, lane 1), and robust RNP formation was observed with WT IEP (Fig. 4D, lane 2). Similar to the observed reduction in RT activity, the mutant IEPs displayed lower affinity for *Ta.it.*I1 RNA. The pulled-down mutant RNP complexes contained hydrolytic RNA splicing products. This demonstrates that RNA coordinated to the mutant IEPs can no longer be stimulated to perform branched splicing (Fig. 4D, lanes 3–4). Although we cannot exclude the possibility that the RNA undergoes hydrolytic splicing prior to RNP assembly, the absence of branched products supports that the proposed DVI–IEP interaction is required for lariat formation. Similar results were observed in bulk IEP splicing assays. WT RNA spliced with WT IEP promoted lariat formation, with ∼4× more lariat produced than lariat-3′ exon splicing intermediate (Fig. 4E, lane 3). IEPs containing K to E charge flip mutations were unable to form lariats, resulting in the accumulation of hydrolytic splicing products (Fig. 4E, lanes 4–5).

To further define the IIC branched splicing reaction, we examined the role of the DII–DVI π–π′ interaction by mutating the DII π GNRA tetraloop to a UUCG loop that is unable to participate in tetraloop docking interactions (Fig. 1). Mutation of the π tetraloop caused accumulation of the lariat-3′ exon splicing intermediate in IEP-dependent splicing reactions (Fig. 4E; lane 8). This phenotype is similar to effects observed for the same mutation in group IIB introns (Robart et al. 2014). Stalling between the two steps of hydrolytic splicing was also observed for the π mutant in the absence of IEP. Thus, the π–π′ interaction generally promotes the second step of group IIC splicing regardless of the attacking nucleophile used in the first step of splicing. Interestingly, the π mutant formed lariat product in the absence of IEP or in the presence of mutant IEPs that failed to support splicing of the WT intron (Fig. 4E; lanes 7,9,10). Thus, the π–π′ interaction also functions to inhibit branching reactions in the absence of IEP. The π tetraloop mutant intron splicing was compared to the WT intron in a time-course splicing assay (Fig. 4F). Both the WT and π mutant have a fast-initial burst, converting ∼50% of the precursor into branched splicing products after 15 seconds incubation in the presence of IEP. At short incubation points, the π mutant produced primarily the lariat-3′ exon intermediate, highlighting the important mechanistic function of the π–π′ interaction in the transition between the first and second steps of splicing. As the reaction was allowed to proceed, the stalled first step lariat-3′ exon splicing intermediate was slowly converted to the second step lariat product.

### Retrohoming assays into fluorescently labeled DNA target sites

**FIGURE 5.**
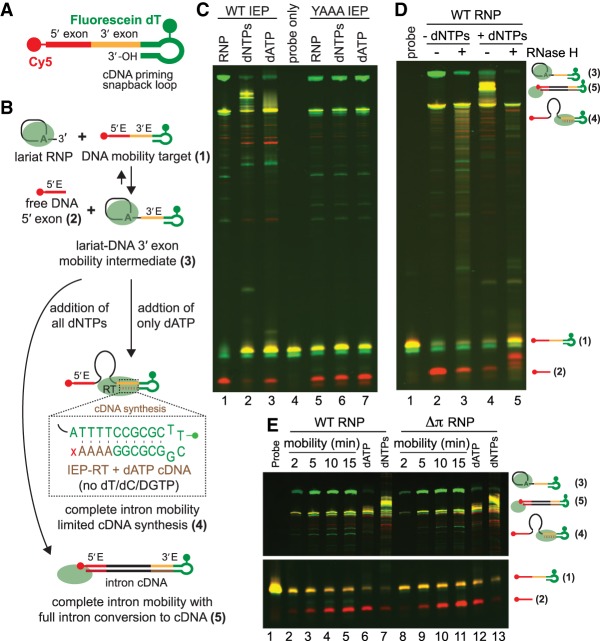
*Ta.it.*I1 in vitro retromobility. (*A*) Cartoon representation of the fluorescently dilabeled DNA substrate engineered for fluorescent in vitro retromobility assays. (*B*) Schematic diagram of reverse splicing and cDNA synthesis. Each expected intermediate and product are numbered in the order they appear in the reaction. The dashed line box insert shows a close-up view of the short intervening sequence containing a polydT stretch engineered to allow limited cDNA synthesis with dATP addition. (*C*) WT and YAAA mutant *Ta.it.*I1 RNP retromobility into DNA substrate; assay was performed with or without addition of dATP/dNTPs. Pseudocoloring of product bands on the gel correspond to which fluorophore was detected: Cy5 = red, FAM = green, both = yellow. (*D*) RNase H digestion of mobility products confirms the presence of extensive cDNA synthesis. (*E*) Time-course of WT and π mutant *Ta.it.*I1 RNP retromobility into DNA substrate.

The IEP splicing reaction produces a lariat-IEP RNP complex that is the entry molecule for group II intron retromobility reactions (Saldanha et al. 1999; Matsuura et al. 2001). During mobility, introns invade new DNA locations by reverse splicing followed by conversion of the intron to cDNA by the RT domain of the IEP. To test retrohoming activity of the *Ta.it.*I1 RNP, we designed a mobility assay utilizing a fluorescent oligo probe as DNA substrate. The substrate contains a 5′ end Cy5 labeled DNA stem–loop that mimics a transcriptional terminator followed by an IBS1 element, and a 3′ end hairpin snapback loop labeled internally with fluorescein dT (FAM) to prime cDNA synthesis (Fig. 5A). This fluorescent probe was designed to allow each intermediate and product to be differentially labeled and detected (Fig. 5B). A short stretch of poly dT was engineered into the section between the 5′ exon and 3′ snapback loop allowing us to control the extent of cDNA synthesis (Fig. 5B, inset). Addition of dATP alone into the mobility reaction allows only 4 nt of cDNA synthesis to occur. In the presence of all dNTPs, cDNA synthesis is not controlled, allowing the entire *Ta.it.*I1 intron to be unwound and used as template by the RT (Fig. 5B).

To assay *Ta.it.*I1 RNP mobility, we allowed the RNP to assemble using branched splicing reaction conditions (described above). DNA substrate was then added to the RNP product in the presence or absence of dNTPs and incubated at 37°C. Incubation of the RNP in the absence of dNTPs primarily produced the lariat-3′ DNA exon mobility intermediate and free 5′ DNA exon products (Fig. 5C, lane 1). Allowing a short stretch of cDNA synthesis by incubation with dATP, but not the other three deoxynucleotides, shifted the reverse splicing reaction to completion, forming fully integrated intron product (Fig. 5C, lane 3). Similarly, addition of all four dNTPs stimulated complete intron integration into the DNA target site, and allowed synthesis of full-length cDNA product (Fig. 5C, lane 2). The full-length cDNA product is larger than the full-length integration product due to covalent linkage of the cDNA product primed by the 3′ exon priming snap back loop to the integrated DNA-intron RNA (Fig. 5A). To confirm the identity of the cDNA product produced in reactions containing all four dNTPs, the reactions were digested with RNase H, which degraded the nascent cDNA-intron RNA hybrid (Fig. 5D, lane 5). We further tested the mobility of RT active site mutant (YADD to YAAA) RNP. The RT mutant displayed WT activity for RNP formation and first step reverse splicing activity. However, addition of dNTPs was not capable of stimulating complete reverse splicing due to inhibition of cDNA synthesis (Fig. 5C, lanes 5–6). Together, our data demonstrate that productive engagement of the RT domain of the IEP in cDNA synthesis is closely linked with the second step of *Ta.it.*I1 reverse splicing.

We demonstrated that the *Ta.it.*I1 group IIC intron uses a π–π′ interaction to position the branch point during the forward intron splicing reaction (see above). We next investigated if the π–π′ interaction is also important for the progression of the reverse splicing reaction into DNA substrates. We did not observe any differences in reverse splicing activity of mutant RNPs compared to WT (Fig. 5E). There were no significant differences in the accumulation of full reverse splicing intron integration after dATP stimulation or cDNA synthesis after dNTP stimulation (Fig. 5E, lanes 6–7 and 12–13). Thus, mutation of the π–π′ contact with the branch point creates a stall between the two steps of forward splicing but does not significantly impair reverse splicing or cDNA synthesis during *Ta.it.*I1 integration into DNA substrates.

## DISCUSSION

In this study, we show that the *Ta.it.*I1 group IIC intron from the thermophilic bacterium *Thermoanerobacter italicus* is dependent upon its encoded IEP for lariat formation. Competition between the hydrolytic and lariat forming splicing pathways is starkly different between group II intron structural classes. While many IIA and IIB intron ribozymes can readily position the bulged adenosine to promote branched splicing, many IIC introns appear to have lost this RNA mediated coordination interface, and default to hydrolysis (Granlund et al. 2001; Toor et al. 2006, 2010; Mohr et al. 2018). Interestingly, chimeric IIC introns combining the *O.i.* intron with features from one of the few reported group IIC introns capable of catalyzing branched splicing reactions (*A.v.I2* from *Azotobacter vinelandii*) could switch splicing preference from hydrolysis to branching (Monachello et al. 2016). In these IIC chimeras, lariat formation was favored over hydrolysis by adjusting the spacing of the bulged adenosine in DVI, restoration of the DII–DVI interface, and substitutions in the DIC stem. Cumulatively, these chimeras reverse-engineered a IIB-like DVI interaction network. Group IIB intron cryo-EM studies have expanded the DVI coordination network to include a combination of DIC–DVI RNA–RNA interactions (ι–ι′) and IEP–DVI protein–RNA interactions (Haack et al. 2019). Together, these studies support a model where lariat forming group IIA and IIB ribozymes position the bulged adenosine first step nucleophile through an RNA mediated DIC–DVI interface via the ι–ι′ interaction in the absence of IEP (Li et al. 2011; Monachello et al. 2016). Group IIC introns generally lack this interface and cannot position the bulged adenosine nucleophile in ribozyme reactions alone, causing the hydrolytic pathway to prevail. To compensate for this missing interaction our data, along with other group IIC reconstitution studies, show that the IEP is chiefly responsible for coordination of DVI through IEP–RNA interactions. We show that a patch of basic amino acids within the maturase domain of *Ta.it.*I1 IEP are required for lariat formation, and propose that these positions form an IEP–DVI binding interface in group IIC introns similar to structural observations in IIB introns.

**FIGURE 6.**
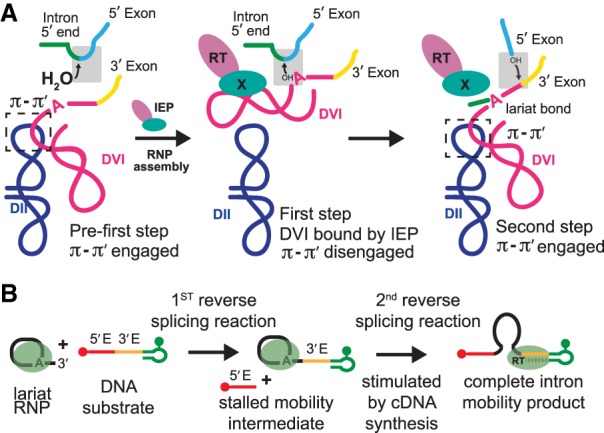
Transitions between the steps of group IIC intron splicing and mobility. (*A*) Forward splicing. Pre-first step, hydrolytic reactions are promoted by sequestration of DVI by π–π′. RNP assembly with the IEP dissociates π–π′, allowing DVI coordinating through IEP interactions (IIB intron: PDB 6MEC). Transition between the steps of forward splicing reforms π–π′ (PDBs 4R0D, 6ME0, 5G2X). This removes the branchpoint from the active site (gray box) allowing the second step to proceed. (*B*) Reverse splicing during mobility. The first mobility step accumulates intron lariat-DNA 3′ exon product. Stimulation of cDNA synthesis by dNTP addition promotes the second step of reverse splicing through IEP conformational changes. Predicted DVI dynamics are panel *A* in reverse.

Structural studies of IIB introns revealed that DII plays an essential role in the transition between the two steps of forward splicing mediated by the π–π′ and η–η′ RNA tetraloop interactions between DII and DVI (Chanfreau and Jacquier 1996; Robart et al. 2014). Although biochemical studies of chimeric group IIC introns tentatively inferred a π–π′ interaction, structural studies used a truncated DII that facilitated crystallization but prevented direct visualization of this interaction (Toor et al. 2008; Costa et al. 2016; Monachello et al. 2016). Here, we show that IIC introns coordinate the lariat bond during the transition between two steps of IEP-dependent splicing through the π–π′ interaction (Fig. 6A). Mutation of the DII π GNRA tetraloop stalls the progression of IEP dependent splicing reactions, resulting in the accumulation of lariat-3′ exon splicing intermediate. Impaired splicing was also observed in hydrolytic reactions in the absence of the IEP, demonstrating that the π–π′ interaction enhances the efficiency of the second step of IIC intron splicing.

Disruption of the π–π′ interaction resulted in low levels of lariat formation in the absence of the IEP. This indicated that the function of the π–π′ interaction in IIC introns is twofold: promoting the progression through the steps of forward splicing, and inhibiting branching in the absence of the IEP. The π–π′ interaction anchors DVI to DII; thus, mutation of this interface would be predicted to allow DVI to explore multiple conformations including transient coordination to the active site. Additionally, incubation of the π mutant with WT IEP stimulated the splicing reaction, illustrating that the IEP–DVI interface is required for efficient initiation of the forward branching reaction. The dynamics of DVI between its π–π′ and IEP-bound conformations provides a framework to mechanistically understand group IIC RNP assembly. We speculate that in the absence of the IEP, DVI may dock into DII via the π–π′ interaction *immediately* after transcription, sequestering the bulged adenosine far from the catalytic site (Fig. 6A). This scenario provides a rationale for why group IIC introns default to hydrolytic reactions in the absence of their IEP. Upon assembly with the IEP, the ribozyme would be activated for lariat formation by undocking π–π′, and handing DVI off to the IEP in a large conformational change. This RNA-to-RNP DVI transition would allow the bulged adenosine access to the active site and trigger the forward branched splicing reaction (Fig. 6A). We attempted to validate this model through FRET assays but were unsuccessful due to loss of activity upon integration of bulky dye adjuncts near the bulged adenosine.

We extended our splicing studies to examine the transition between the two steps of reverse splicing during intron mobility into fluorescently labeled DNA substrates. The *Ta.it.*I1 IIC intron is a highly active retroelement that rapidly integrates into DNA by completion of the first step of reverse splicing. The π mutant RNA showed an impairment between the two steps of forward splicing; however, we observe no effects of the π mutation in mobility reactions. During the transition between the steps of forward splicing, the active site is reorganized through exchange of catalytic triplex conformations (Chan et al. 2018). Although lariat formation is impaired by the π mutation, ribozymes that successfully complete the forward splicing reaction would be preconfigured in a catalytic triplex arrangement to immediately perform the analogous reverse splicing step.

We, as well as others, observed that in the absence of dNTPs intron mobility accumulates the lariat-3′ DNA exon mobility intermediate. Upon addition of dNTPs to the mobility reaction, the mobility intermediate completes the second step of reverse splicing, fully integrating the intron into DNA substrates. Thus, engagement of the RT domain of the IEP is the major mechanistic control point for the transition between the two steps of reverse splicing. This finding mirrors mobility observations for the *LtrA/L.l.LtrB* group IIA intron RNP (Aizawa et al. 2003; Mastroianni et al. 2008). Priming of cDNA synthesis varies between different group II introns. Introns whose IEP contains an endonuclease domain nick the opposite strand of DNA targets to create a primer in the target site, which is used to integrate the inserted intron by cDNA synthesis (Zimmerly et al. 1995). Group IIC introns, as well as the RmInt 1 group IIB intron, lack this endonuclease activity, and instead rely upon nascent replication forks to prime cDNA synthesis (Zhong and Lambowitz 2003; Martínez-Abarca et al. 2004; García-Rodríguez et al. 2019). At this time, it is unclear what conformational changes in the RNP facilitate the completion of the second step of reverse splicing; however, it is very likely that priming cDNA synthesis is closely coupled to the 5′ DNA exon attack of the lariat bond.

## MATERIALS AND METHODS

### Plasmids

*Ta.it.*I1 IEP expression plasmid and mutations were synthesized by Genscript. A codon optimized fusion of 8xHis-SUMO-*Ta.it.*RT was cloned into pET11a by NdeI and BamHI to produce pET11a-His-SUMO-TaitRT. *Ta.it.*I1 in vitro transcription templates were also made by Genscript. Plasmids used in the study are freely available from MolecularCloud.

### Expression and purification of *Ta.it*.I1 IEPs

pET11a His-SUMO-*Ta.it*.RT and its mutants were expressed in Rosetta (DE3) *E. coli* cells. Expression was performed with 1.5 L of Terrific broth auto-induction media (Studier 2005) containing 100 µg/mL carbenicillin and 20 µg/mL chloramphenicol grown at 25°C for 24 h. Cells were harvested at 5000*g* and resuspended in a final volume of 40 mL of lysis buffer (20 mM Tris-HCl [pH 7.5], 300 mM NaCl, 5 mM β-mercaptoethanol, 10 mM imidazole) and treated with PMSF protease inhibitor at a 1/1000 dilution. The cells were lysed on ice by sonication: 15 bursts of 8 sec separated by 1-min rests. The cell lysate was cleared at 25,000*g* at 4°C, added to 2 mL Ni-IDA agarose beads (GoldBio) and allowed to bind in a conical tube while turning end-over-end for 30 min at 4°C. The resin was collected at 500*g* at 4°C and washed with 30 mL of lysis buffer. This process was repeated three times to preclean the resin prior to column loading, which was observed to significantly reduce RNase contamination of the IEP preparation. After prewashing, the resin was loaded onto a 20 mL column, washed with 30 mL of wash buffer (20 mM Tris-HCl pH 7.5, 300 mM NaCl, 5 mM β-mercaptoethanol, and 20 mM imidazole), followed by a wash with 2 M NaCl, 20 mM Tris-HCl (pH 7.5), 5 mM β-mercaptoethanol to remove copurifying nucleic acids bound to the IEP. The YAAA mutant IEP was particularly prone to copurification with non-specific nucleic acids judged by OD 260 nm, which may have caused higher background in PicoGreen RT assays (see below). After rinsing the resin with lysis buffer, the protein was eluted with ∼15 mL of elution buffer (20 mM Tris-HCl pH 7.5, 300 mM NaCl, 10 mM β-mercaptoethanol, and 300 mM imidazole). The IEP was desalted/buffer exchanged in desalting buffer (0.2 M NH_4_Cl, 10 mM MgCl_2_, 50 mM Tris-HCl pH 7.5, 5 mM β-mercaptoethanol) on a HiPrep Desalting FPLC column (AKTA). IEP was further purified by size exclusion chromatography using a Sephacryl S-200HR column. The final protein was concentrated at 4000*g* followed by addition of 5 mM DTT and glycerol to a final concentration of 50%. The IEP was stored at −20°C.

### Fluorescent reverse transcriptase (RT) activity assays

*Ta.it.*I1 IEP was diluted 1:5, 1:10, 1:20, 1:50, and 1:100 in dilution buffer (50 mM Tris-HCl pH 7.5, 2 mM DTT, 20% glycerol). An amount 5 µL of each RT dilution was added to 20 µL of poly(rA) oligo(dT)_18_ substrate and incubated at 25°C for 30 min before quenching with 2 µL of 220 mM EDTA. An amount of 12.5 µL of each reaction was then transferred to one well of a 96 well plate where 86.5 µL of Picogreen reagent was added. An amount of 12.5 µL of dilution buffer was used as a negative control. Fluorescence was measured with a Synergy H4 Hybrid plate reader (BioTek). To normalize mutant *Ta.it.* RT activity to WT *Ta.it.* RT activity, the concentration of each RT was determined by a Bradford assay and each were diluted to 800 µg/mL. RT reactions were set up with 5 µL of 800 µg/mL RT (WT or mutant), to 20 µL of poly(rA) oligo(dT)_18_ substrate, and 2 µL of RNaseA and performed as described above.

### In vitro transcription of *Ta.it.*I1 RNA

RNA transcription was performed with sequence confirmed plasmid DNA and T7 RNA polymerase (Wiryaman and Toor 2017). During transcription optimization, the 10× T7 buffer was replaced with a *Ta.it.*I1 specific 10× T7 buffer containing 5 mM MgCl_2_. After transcription and subsequent DNase I and Proteinase K digestion, the transcripts were filtered, concentrated, and buffer exchanged by several rounds of centrifugation at 4000*g* using an Amicon 100 kDa cutoff ultrafiltration centrifugal filter unit.

### In vitro splicing assays

Standard splicing assays were performed with 1 µg of *Ta.it.*I1 WT RNA, 5 mM fresh DTT, 50 µL of splicing buffer (10 mM MgCl_2_, 50 mM Tris-HCl [pH 7.5], 0.5 M NH_4_Cl final concentration) and made up to 100 µL volume with Millipore water. Splicing reactions were performed by preincubation of the splicing buffer with or without IEP for 5 min at the assay temperature and initiated by addition of the RNA. Hydrolytic splicing assays were performed without IEP under 100 mM MgCl_2_ buffer conditions. Maturase splicing assays were performed with ∼1.5× molar excess of *Ta.it.*I1 IEP. Reactions were phenol-CIA extracted and ethanol precipitated with 0.3 M NaOAc, pH 5.2 with a linear polyacrylamide carrier. Extracted RNA was resuspended in water and 2× formamide/2 mM EDTA loading buffer before heating at 95°C for 3 min. Samples were resolved on 4% polyacrylamide (19:1 acrylamide:bisacrylamide)/7 M urea gels, stained with ethidium bromide, and imaged on the Syngene G:Box imaging system.

### IEP pull-down assay

Ni-IDA-agarose beads (GoldBio) were blocked with 100 ng/µL BSA and 1 µM pMALc5x-Rev oligonucleotide in 10 mM MgCl_2_, 250 mM NH_4_Cl, 50 mM Tris-HCl (pH 7.5) buffer. A 10 µg *Ta.it.*I1 RNA IEP-assisted splicing reaction in pull-down buffer: 250 mM NH_4_Cl, 10 mM MgCl_2_, 1 mM β-mercaptoethanol, 50 mM Tris-HCl (pH 7.5). The reaction was added to the blocked beads and allowed to bind end-over-end at room temperature for 30 min. The resin was collected by centrifugation at 500*g* for 2 min and washed with 500 µL of pull-down buffer at room temperature for 15 min three times. Pulled-down RNPs were eluted with pull-down buffer supplemented with 300 mM imidazole. Eluted RNPs were phenol-CIA extracted, ethanol precipitated with 0.3 M NaOAc, pH 5.2 with a linear polyacrylamide carrier, resolved on 4% polyacrylamide/7 M urea gel, and stained with ethidium bromide.

### Fluorescent oligonucleotide substrate

Mobility assays were performed with multiple different oligonucleotides labeled with fluorescent probes to eliminate the need for radioactivity. DNA substrate was designed with a 5′ end DNA stem–loop labeled with Cy5 and a 3′ end hairpin labeled with FAM. The sequence of the substrate, synthesized by IDT, was: Cy5GCAGTCTAAAAGTAATTTTAGACTGCTTTTTTATTTTTTCCGCGCTTCGGCGCGG, underlined position indicates the internal FAM label position.

### Fluorescent in vitro mobility assays

RNPs for mobility reactions were assembled by IEP-dependent splicing reactions under standard maturase conditions stated above at 37°C for 15 min. Mobility reactions were performed with 500 nM substrate with or without 1 mM dNTPs or dATP. Controls included were substrate alone and reactions treated with 50 U RNase H. All reactions and controls were incubated at 37°C for 15 min before phenol-CIA extraction and ethanol precipitation with 0.3 M NaOAc (pH 5.2). Reactions at higher temperatures did not support mobility, likely due to melting of the stem–loops in the DNA substrate. Time course assay reactions were stopped using stop solution (0.3 M NaOAc, 20 mM EDTA, 200 µL phenol-CIA). Extracted RNA was resuspended in water and 2× formamide/2 mM EDTA loading buffer before heating at 95°C for 3 min. Samples were then resolved on 4% polyacrylamide/7 M urea gels and scanned for fluorescence on a Typhoon imaging system (GE Amersham).
